# Multidisciplinary brain metastasis clinic: is it effective and worthwhile?

**DOI:** 10.3332/ecancer.2020.1136

**Published:** 2020-11-05

**Authors:** Annu Rajpurohit, Nilendu Purandare, Aliasgar Moiyadi, Prakash Shetty, Abhishek Mahajan, Rajiv Kumar, Subhash Yadav, Naveen Munmudi, Ameya Puranik, Anil Tibdewal, Rahul Krishnarthy, Ankita Ahuja, Nandini Menon, Vanita Noronha, Amit Joshi, Vijay M Patil, Kumar Prabhash

**Affiliations:** 1 Department of Medical Oncology, Tata Memorial Hospital, Homi Bhabha National Institute (HBNI), Mumbai 400012, India; 2 Nuclear Medicine, Tata Memorial Hospital, Homi Bhabha National Institute (HBNI), Mumbai 400012, India; 3 Neurosurgery, Tata Memorial Hospital, Homi Bhabha National Institute (HBNI), Mumbai 400012, India; 4 Radiodiagnosis, Tata Memorial Hospital, Homi Bhabha National Institute (HBNI), Mumbai 400012, India; 5 Pathology, Tata Memorial Hospital, Homi Bhabha National Institute (HBNI), Mumbai 400012, India; 6 Radiation Oncology, Tata Memorial Hospital, Homi Bhabha National Institute (HBNI), Mumbai 400012, India

**Keywords:** multidisciplinary, brain metastasis clinic, targeted therapy, intrathercal chemotherapy

## Abstract

**Background:**

Management of brain metastasis is a complex multidisciplinary venture. Hence, we started a multidisciplinary brain metastasis clinic for the opinion on difficult brain metastasis cases. This is the review of the impact of this clinic on the treatment decisions.

**Methods:**

The brain metastasis clinic (BMC) was started in April 2018 and meets once a week. Data of patients discussed between 27th April 2018 and 28th June 2019 were included for this analysis. Treatment decision made by clinicians (before sending the patient to the BMC) was compared with the decisions made in BMC. The decisions were broken on a predefined proforma as the intent of treatment (curative or palliative), modalities planned (surgery, radiation, chemotherapy) and type of therapy planned (details of each therapy) in each modality were collected both pre and post BMCs. In addition, compliance of the respective physicians to BMC decision was also calculated. SPSS version 20 was used for analysis. Descriptive statistics were performed.

**Results:**

Ninety-nine patients were discussed in this time period. The median age was 51 (range 17–68) years. The gender distribution was 70 males (70.7%) and 29 females (29.3%). Lung was the predominant site of malignancy (79, 79.8%). Thirty-one patients (31.3%) had EGFR TKI domain activating mutation, while 17 (17.2%) had anaplastic lymphoma kinase (ALK) rearrangement. The treatment plan was changed in 46 patients (46.5%). The intent of treatment was changed from palliative to curative in 5%. Change in the treatment plan with respect to surgery in 9.1%, radiation in 37.4%, chemotherapy in15.2%, targeted therapy in 22.9% and intrathecal in 6.1% patients, respectively. The compliance with the BMC decision in patients in whom it was changed was 84.8% (39, *n* = 46).

**Conclusion:**

Multidisciplinary management of difficult brain metastasis cases in specialised clinics has a significant impact on treatment decisions.

## Introduction

Brain metastasis is the most common malignancy of the central nervous system (CNS). Due to the improvement in systemic therapies in solid tumours leading to the improvement in the overall survival of a metastatic disease, there is an increasing incidence of failure in sanctuary sites like CNS metastasis [1]. This is because of the inability of these therapies to achieve adequate cerebrospinal fluid concentrations as traditional cytotoxic chemotherapy has limited blood–brain barrier penetration [2]. Furthermore, certain cancers like driver mutated non-small cell lung cancer (NSCLC) have a predilection for developing brain metastasis [3]. Management of patients with CNS metastasis from solid tumours is a challenge, in terms of diagnosing, selecting appropriate treatment modality and detecting progression [4]. Hence, multidisciplinary team management is required to offer the best possible option for the patient.

Multidisciplinary clinics in cancer management help in the speedy selection of appropriate treatment modalities. We have multidisciplinary joint clinics for the management of broad disease-specific sites; however, BMC for an opinion on difficult brain metastasis cases was conspicuously missing. Hence, recently a stage-wise plan to roll out a BMC was formed and implemented in our hospital. One of the objectives of the first stage of this multistage plan was to provide solutions for the management of brain metastasis patients who had diagnostic dilemmas and to help select the most appropriate treatment modality. This is the audit of the initial consecutive 99 patients who were discussed in this clinic to estimate the impact of BMC on treatment decisions of these patients.

## Patients and methods

### Development and functioning of BMC

The BMC was developed to provide comprehensive care in patients with brain metastasis. The lead author was entitled to the responsibility of staring at the clinic. BMC was started in the month of April 2018 and meets once a week. The clinic accepts cases all days. The clinical history, physical examination and radiological records are examined and a short summary is prepared for each patient. The intent, treatment plan and the question of the referring physician is noted. Any extra investigations, if felt necessary are done prior to keeping the case in the multidisciplinary meeting. During the post discussion in the multidisciplinary clinic, the team suggests the intent and treatment plan for each patient which is subsequently conveyed to the referring physician.

Appendix 1 shows a typical report highlighting all the discussion in BMC.

### Data collection for current audit

Data of patients discussed between 27th April 2018 and 28th June 2019 were included for this analysis. The data regarding age, gender, diagnosis, mutation profile, previous treatment details brain metastasis details, preclinic treatment plan and post clinic treatment plan were noted as shown in Table 1. This data was obtained from the brain metastasis clinic records and was entered in google forms.

### Statistical analysis

The data entered in google forms were converted into google sheet and subsequently were imported into SPSS and R for analysis. In this analysis, descriptive statistics were performed for demographic and tumour characteristics. The continuous variables were expressed as median with interquartile range, while ordinal and nominal variables were expressed as a percentage with 95% CI. The treatment decision made by physicians before sending the patient to the BMC were compared with the decisions made in BMC. The decisions were broken as the intent of treatment (curative or palliative), modalities planned (surgery, radiation, chemotherapy), and type of therapy planned in each modality. For example, in a patient with multiple brain metastases, intent would be palliative – the modalities planned would be radiation and chemotherapy while the radiation therapy type planned would be whole brain radiotherapy.

The intent of treatment, the number of patients planned for each modality and type of modality were compared between pre and post BMC. The number (and %) of patients whose treatment plan was modified was estimated.

## Results

A total of 99 patients were discussed in BMC from 27^th^ April 2018 to 28^th^ June 2019. The median age of the patients was 51 years (17–68), 70.7% were males and the majority of the patients had lung cancer (79.8%). Out of 79 lung cancer patients, 31 (39.2%) patients were EGFR mutated and 17 (21.5%) had anaplastic lymphoma kinase (ALK) mutation. Brain metastasis was not confirmed in 15 (15.1%) patients and was kept as a differential diagnosis before BMC. The overall treatment plan was altered as a result of discussion in BMC in 46 (46.4%) patients. Six patients were previously treated for brain metastasis (two patients underwent metastasectomy and four patients had received radiation therapy).

Out of 94 patients planned for palliative intent therapy before BMC, the treatment intent changed to curative in 5 (5.3%) patients. Six (6.2%) patients out of 96 patients planned for non-surgical intervention were planned for surgical management in BMC. The reason for the change of plan to surgical intervention in all the patients was the identification of oligometastatic brain disease and reclassification of the suspected metastatic lesion as indeterminate for metastasis.

The criteria for change in treatment were the primary tumour with radical intent treatment (either surgery or chemoradiation) with single or oligometastatic brain metastasis , and they underwent either surgery or stereotactic body radiation therapy (SBRT) for brain metastasis after discussion in multi-disciplinary brain clinic. Out of five patients where curative treatment offered, two are disease-free at the follow-up of 24 months. Three patients had a progressive disease where the next line treatment has started, and one out of three died of disease progression.

Out of 20 patients who were not planned for radiotherapy, 10 (50%) were planned for radiotherapy in BMC. The most common reason (70%) for consideration of radiotherapy was asymptomatic progression in brain metastasis in patients on TKI. Another reason (20%) was oligometastatic lesions in the brain in a critical area where surgery was considered to be difficult and thus, planned for radiotherapy in BMC. Another reason (10%) for radiotherapy was the better characterisation of the lesion in the brain in BMC which was previously considered of doubtful aetiology. Out of 79 patients who were planned to be given radiotherapy pre-BMC, 27 (34.2%) patients were planned not to be given radiotherapy in BMC. The most common reason (74%) for such a change in treatment decision in BMC was an asymptomatic lesion in non-critical area of the brain. Another important reason (7.4%) was the characterisation of brain lesions to be of benign aetiology (neurocysticercosis).

Out of 46 patients planned for chemotherapy pre-BMC, 13 (28.3%) patients were planned not to receive chemotherapy in BMC. The reason for changing the plan of chemotherapy in BMC in all the patients was oligoprogression in the brain in patients on TKI which was targeted with radiotherapy and TKI was continued beyond progression. On the other hand, out of 53 patients who were planned not to receive chemotherapy pre-BMC, 2 (3.8%) were planned to be given chemotherapy in BMC. The reason for the change in treatment decision was the establishment of progression in brain lesions in patients on TKI with no feasibility of second-line TKI. Out of 48 patients who were planned for discontinuation of targeted therapy and shift to chemotherapy in view of progression, 11 (22.9%) patients were planned to continue the same targeted therapy.

Out of 98 patients who were not planned to receive intrathecal treatment, 6 (6.2%) patients were planned to receive intrathecal chemotherapy in BMC. The diagnosis of brain metastasis was modified to benign lesions in 5.3% of patients. The compliance with the BMC decision in patients in whom it was changed was 84.8% (39, *n* = 46).

## Discussion

The results of our analysis confirm the importance of BMC. The metrics to measure the success of multi-disciplinary brain clinics are changes in treatment decisions and intent. The treatment plan was overall modified in 46 per cent of patients. The modification was predominantly in the selection and type of treatment modality. These changes suggest the need for a dedicated multidisciplinary clinic for dealing with brain metastasis patients as these patients had already been through a site-specific multidisciplinary clinic. There has been very limited data on the outcomes and treatment changes suggested by BMC. Mckee et al. published data of 65 breast cancer patients treated as per recommendations in BMC [5].

The results need to be interpreted in the context of the BMC referral pattern. The referral was made not for all consecutive patients with brain metastasis seen in the institute but in those patients in whom the referring physician had some dilemma. Hence, these results pertain to this cohort of patients. The change in the decision in treatment modality is to a large extent reflects stereotactic radiotherapy (SRT) and intrathecal chemotherapy being more commonly recommended by the BMC than the referring physician. The underutilisation of SRT in the current study appears to be due to a lack of understanding of the prognosis of driver-mutated NSCLC by the treating radiation oncologist. Besides, patient logistics, high patient burden and financial status are other important reasons for the limited use of SRT.

The median OS of driver mutated patients even in brain metastasis patients is in the range of 3–5 years [6]. Even in non-driver-mutated NSCLC with PDL1 expression of > 1 per cent the median OS is in the range of 15–25 months, with 20–30 percent of patients having potential for survival beyond 3–5 years [7]. These results are better than those seen with potentially curative multimodality treatment seen in pancreatico-biliary malignancies and esophageal malignancies. Hence, all efforts must be made to prolong survival and preserve the cognitive functions of these NSCLC patients. Similar improvement in survival was seen in breast cancer patients [8].

An underutilisation of intrathecal chemotherapy by referring physician was seen [9]. This is likely due to the limited evidence of intrathecal chemotherapy in the situation of leptomeningeal metastasis [10]. The dural involvement in imaging may predict the possibility of involvement of CSF, and in such cases, as there is inter radiologist variation in diagnosis, intrathecal chemotherapy may be useful [11, 12]. The results seen in the audit were obtained on patients from a tertiary cancer centre and, hence, are generalisable. The results had their own limitation as most of these patients had primary NSCLC, and as there was underutilisation of SRT, future studies with a larger patient population are required. The referring physicians were experienced and qualified consultants in their respective fields. However, it should be noted that the patients being treated in a non-specialised centre may derive greater benefit from such a clinic as the expertise to treat difficult cases may not be available.

The advantage of this clinic over primary tumour multidisciplinary clinic is that there are neurology dedicated oncologists, pathologists, radiologists and neurosurgeons at the same time for detailed clinical discussion for precise decisions, and it ultimately saves time and improves patient care with brain metastasis. This clinic also has disadvantages as it only happens once a week, logistics related to all specialists sparing time in their busy hospital schedule and logistics related to the patients. The average time between BMC clinic decision and intervention is one week, so patients who require urgent intervention cannot be discussed in this clinic and required interventions are done on an urgent basis by each speciality after discussing with the primary unit.

## Conclusion

Multidisciplinary management of difficult brain metastasis cases in specialised clinics has a significant impact on treatment decisions.

## Figures and Tables

**Table 1.** Baseline characteristics of study patients and treatment planned in brain metastasis clinic.

Variable	Value
Total patients	99
Time period	27th April 2018 to 28th June 2019
Median age (years)	51 (17–68 years)
Gender Male Female	70 29
Performance status 0–1 2	90 9
Diagnosis Lung cancer Renal cell carcinoma Testicular carcinoma Breast cancer Others	79 08 01 03 08
Driver mutation status in lung cancer EGFR mutation ALK rearrangement	31 17
Previous treatment for primary Surgery Radiation Chemotherapy	03 79 46
Number of brain metastasis Single Multiple	40 59
Prior treatment for brain metastasis Surgery Radiotherapy	2 4
Brain metastasis^a^ Confirmed Non confirmed	84 15
Treatment planned in BMC Surgery Surgery + Radiotherapy WBRT SRS	4 2 49 13

a This is the opinion on radiology of the referring physician.

## Appendix 1

**Brain metastasis clinic - 12.10.2018**

**Patient ID – XYZ**

**Age/Gender – 53/Female**

**Case summary** – Evaluated and started on treatment **outside TMH**.

53 years old lady with no comorbidities diagnosed as Metastatic adenocarcinoma lung in January 2016.

Metastatic sites – B/L lung nodules , pleura , bones )

EGFR /ALK –negative PD L1 – not done

Shes was started on Pemetrexed + carboplatin , received 4 cycles ( till march /2016) and had partial radiological response. After this maintenance Pemetrexed was started ,received 6 cycles and switched to tab Gefitinib 250 mg OD in august /2016.

In October /2017 – clinical and radiological progression (increase in lung nodules + new onset bone metastasis).

**Referred to our hospital**

Repeat lung biopsy was done.

Histopathology report - Adenocarcinoma

RT -PCR – Exon 19 deletion & T 790M mutation

Patient was not affordable for Osimertinib , so rechallenged with 4 # Pemetrexed + carboplatin >> followed by pemetrexed maintenance till October /2018.

Then presented with history of Giddiness , slurring of speech and right sided weakness.

MRI Brain was showing multiple metastasis in bilateral cerebrum and cerebellum.

**Patient was referred to brain metastasis clinic**

On examination

Higher mental function – normal

Right sided upper and lower limb motor weakness – 4/5

Sensory examination – normal , Bowel /bladder – normal

Cerebellar signs – present

IMPRESSION – Metastatic Adenocarcinoma lung –EGFR 19 and T790M mutation+ with new onset multiple brain metastasis

**BMC clinic decisions -**

Supportive care

WBRT - 20 Gy / 5 #

CSF Cytology and EGFR testing

Restaging CECT Thorax + abdomen >>stable disease

Patient was started on WBRT and CSF cytology was positive for malignant cells. RT PCR EGFR in CSF showed T790 M mutation.

**BMC Rediscussion and decisions-**

Tab Osimertinib 80 mg OD (through Patient support program )+ Intrathecal methotrexate
